# Role of Anaesthetic Choice in Improving Outcome after Cardiac Surgery

**DOI:** 10.2478/rjaic-2020-0017

**Published:** 2020-12-31

**Authors:** Mihai Stefan, Daniela Filipescu

**Keywords:** cardiac anaesthesia, cardiac surgery, total intravenous anaesthesia, volatile anaesthetics, anaesthetic preconditioning, myocardial ischaemia

## Abstract

**Clinical background:**

Volatile anaesthetics (VAs) have been shown to protect cardiomyocytes against ischaemia and reperfusion injury in cardiac surgery.

**Clinical problems:**

VAs have been shown in multiple trials and meta-analyses to be associated with better outcomes when compared to intravenous anaesthesia in cardiac surgery. However, recent data from a large randomised controlled trial do not confirm the superiority of VA as compared to total intravenous anaesthesia in this population.

**Review objectives:**

This mini review presents the VA cardioprotective effects, their clinical use in cardiac surgery and the most recent evidence that compares VA to intravenous anaesthesia for reducing perioperative morbidity. At present, there is no clear superiority of VA over intravenous anaesthesia in improving the outcome after cardiac surgery.

## Introduction

Adult cardiac surgery is associated with significant postoperative morbidity and mortality.[1] Even complications that are considered less severe may cause discomfort to the patient and increase the costs for their treatment. Consequently, the rate of postoperative complications provides more information about hospital quality than in-hospital mortality rates. Considering the potentially avoidable nature of a number of these postoperative complications, preventive methods should be employed to improve the outcome after cardiac surgery. One intervention that may result in better perioperative outcome is the choice of the anaesthetic regimen.[2] As several clinical trials conducted in patients undergoing coronary artery bypass grafting (CABG) surgery suggested relevant cardioprotection by volatile anaesthetics (VA), both the American and European guidelines recommend the use of these agents for reducing the risk of perioperative myocardial ischaemia.[3,4] This mini review presents the VA cardioprotective effects, their clinical use in cardiac surgery and the most recent evidence that compares VA to intravenous anaesthesia for reducing perioperative morbidity.

## Volatile anaesthetics’ cardioprotective effects

There is a long-standing belief that in addition to offering adequate depth of anaesthesia and cardiovascular stability, VAs have a protective effect on cardiomyocytes against hypoxic conditions, such as those occurring during cardiopulmonary bypass (CPB) with aortic cross-clamping. [5] The exposure of cardiomyocytes to VA before, during and after aortic cross-clamping for coronary bypass anastomosis triggers multiple signal pathways to “prepare” the cell for the attendant hypoxia, rendering it more resistant to hypoxic stress damage, a process that is called anaesthetic conditioning, a form of ischaemic preconditioning.[6] Ischaemic preconditioning is an adaptive response that protects myocardium by previous transient ischaemic episodes. Cardiac cell survival in an oxygen-deprived environment is mediated by the activation of mitochondrial mechanisms through potassium-channel openers that trigger the preconditioning pathway.[7] Non-pharmacological preconditioning occurs when the blood supply to the heart is blocked, which exposes the heart risk of ischaemia. Pharmacological conditioning through VA triggers enzymatic mechanisms that confer pre- and postconditioning effects, which are independent of the hypnotic properties of the gases.[7] They attenuate apoptosis and necrosis and reduce myocardial dysfunction after ischaemia and reperfusion. The mechanisms involved in cardioprotection account for decreased cytosolic and mitochondrial calcium loading with the final objective of maintaining intracellular homeostasis through the preservation of the mitochondrion and its normal function.[8,9]

Some authors suggested that VA administration throughout surgery appeared to provide superior protective effects compared with administration either before or after CPB.[10] Existing evidence also supports VA postoperative cardioprotective effects for as long as cardiac surgery patients are in the intensive care unit (ICU).[11] Moreover, the protective effects seem to be related to the amount of VA administered. [12]

## Clinical use of VA cardioprotection

The use of VA agents during CPB was described for the first time in 1974.[13] Originally, VAs were vaporised and administered, mixed with oxygen, in the early-generation bubble oxygenators. Today, cardiac surgery interventions are performed with standard membrane oxygenators. Although recommendations are available to guide the practice of CPB and delivery of VAs,[14,15] the inclusion of a vaporiser in the CPB circuit and the delivery of VA to the patient during CPB is not standardised. Despite all the newest CPB machines having appropriate connections for VA vaporisers, perfusionists still need to adapt anaesthetic vaporisers to the bypass circuit as well as the scavenging systems to not pollute the room. Many manufacturers do not specifically mention that vaporisers may be included in their CPB circuit due to strict regulations prohibiting the routine use of VAs through the circuit in some countries. The 10-year experience of Brazilian and Italian cardiac surgery centres that use VAs during CPB was summarised in some published “tricks and tips” of this technique.[16]

Several randomised controlled trials (RCTs) and meta-analyses suggested that VA use in cardiac surgery (in particular, sevoflurane and desflurane), mimicking ischaemic preconditioning, might reduce perioperative myocardial damage, quantified by the level of troponins.^[17, 18, 19]^ Trial sequential analysis (TSA) indicated that the required information regarding the decrease of cardiac troponins by VA was available for on-pump cardiac surgery since 2006.[18] As the effects of VAs on myocardial biomarker release after on-pump CABG are well established, no more studies are needed on this topic.[20] The lower peak of cardiac troponin in patients receiving sevoflurane was also associated with a reduction in the incidence of late adverse cardiac events as compared to the propofol groups.[17] However, whether these findings result in improved clinical outcomes is currently controversial.

## Anaesthetic techniques and outcome

Landoni’s group from Milan was very active in searching for the outcome effects of VA cardioprotection. Although their meta-analyses found a significant lower mortality in patients receiving VA compared to intravenous anaesthesia (21,22), their multicentre RCT performed in patients undergoing high-risk cardiac surgery has not observed any beneficial effect of anaesthesia with sevoflurane and desflurane compared with propofol-based intravenous anaesthesia on the composite endpoint of prolonged ICU stay and mortality (30 days and 1 year) or both.[23] Further meta-analyses found that general anaesthesia with VA compared to total intravenous anaesthesia (TIVA) was associated with major benefits in outcome, including reduced mortality, as well as lower incidence of pulmonary and other complications.[24,25] A meta-analysis including 58 studies with a total of 6105 participants compared the use of inhalation versus intravenous anaesthesia for adults undergoing on-pump or off-pump CABG surgery and found high-quality evidence that sevoflurane reduces death within 180 to 365 days of surgery (on-pump: relative risk [RR] 4.10; 95% confidence interval [CI] 1.42 to 11.79; *p* = 0.009; I2 = not applicable).[25] There was also a statistically significant difference favouring sevoflurane compared to propofol on both inotropic (RR 2.11; 95% CI 1.53 to 2.90; *p* < 0.00001; I2 = 0%) and vasoconstrictor support needed (RR 1.51; 95% CI 1.04 to 2.22; *p* = 0.03; I2 = 0%). In contrast, in a cohort study performed in three university hospitals in Denmark, there were no differences in postoperative short-and long-term outcomes between VA and TIVA.[26]

Despite the conflicting evidence, an updated international web-based consensus conference process identified VA as one of the 11 interventions contributing to increased postoperative survival after cardiac surgery.[27]

In this context, the results from Mortality in Cardiac Surgery Randomized Trial of Volatile Anaesthetics (MYRIAD)[28] were unexpected. This trial was a multicentre, single-blind controlled trial that included patients scheduled to undergo elective CABG in 36 centres from 13 countries. 5400 patients were randomly assigned to an intraoperative anaesthetic regimen that included a VA (desflurane, isoflurane or sevoflurane) or TIVA. The primary outcome of the trial was death from any cause at 1 year. Secondary endpoints were 30-day mortality; 30-day death or non-fatal myocardial infarction (composite endpoint); cardiac mortality at 30 days and at 1 year; incidence of hospital re-admission during the 1-year follow-up period and duration of ICU and hospital stay. Intraoperative anaesthesia with a VA did not result in significantly lowering the number of deaths at 1-year follow up as compared to TIVA (2.8% in the VA group vs. 3.0% in the TIVA group; RR 0.94; 95% CI 0.69 to 1.29; *p* = 0.71). The same was found to be true for outcomes of death at 30 days (1.4% in the VA group vs. 1.3% in the TIVA group). The incidence of adverse events also did not differ significantly between the two groups. No difference was observed for secondary outcomes either. However, there was a reduction in haemodynamically significant myocardial infarction in the VA group (28). It is important to mention that the study included both on-pump and off-pump CABG, that on-pump CABG was performed in 64% of patients, with a mean duration of CPB of 79 minutes, and the study was stopped for futility at the time of the second interim analysis.

Noticeably, two further meta-analyses including the MYRIAD trial arrived at contradictory conclusions regarding the effect of a VA regimen on 1-year mortality after cardiac surgery.[29,30] However, based on the TSA[29] and considering the lack of homogeneity of studies,[30] respectively, both groups of authors concluded that further large RCTs are needed to clarify the issue of superiority of VA as compared to TIVA in CABG patients.

## Summary and future perspectives

Table 1 summarises the relevant outcome studies cited in this review and mirrors the contradictory results published so far regarding the best anaesthetic regimen for CABG patients. Factors such as non-uniform extent of the ischaemic insult, the differences in anaesthesia protocols, surgery type and procedures, the presence of comorbidities, the effects of concurrent medication, etc. may play a role in negative trials.[31] The ischemia–reperfusion injury in cardiac surgery is a too complex phenomenon to be targeted by one single intervention, such as the choice of the anaesthetic regimen. Moreover, ischaemia–reperfusion injury is less frequent in modern cardiac surgery,[32] and future trials on the benefit of VA should address specific populations and procedures at risk for myocardial damage.

**Table 1:** Summary of the outcome studies presented in the article

First author, year of publication (ref. in the text)	Type of study (number of studies)	Type of surgery (number of patients included)	Intervention	Outcome	Comments
**Landoni, 2007 (21)**	Meta-analysis of RCTs (22)	Cardiac surgery (1,922)	Either sevoflurane or desflurane vs. TIVA (mainly propofol)	VA decreased the rate of PMI (2.4% vs. 4.1%) and in-hospital mortality (0.4% vs.1.6%)	Different regimens of VA administration
**Landoni, 2013 (22)**	Bayesian network meta- analysis of RCTs (38)	Cardiac surgery (63% on-pump CABG) (3,996)	VA vs. TIVA (mainly propofol)	VAs were associated with: - reduced mortality at the longest available follow-up (1.3% vs.2.6%) (results also confirmed when only low-risk-of-bias stud- ies, larger studies and CABG studies were included) - reduced MV time - reduced ICU and hospital LOS Bayesian network meta-analysis found that sevoflurane and desflurane (but not isoflurane) were associated with mortality reduction	The survival benefit was significant only when aggregating the three volatile agents together
**Landoni, 2014 (23)**	Multicentre RCT	High-risk cardiac surgery (200)	Sevoflurane vs. propofol	No difference in death, prolonged ICU stay, or both No difference in postoperative cTn re- lease, 1-year all-cause mortality, re-hospitalisations and adverse cardiac events	The study was not powered to detect a difference in mortality at 30 days and at 1-year follow-up
**Uhlig, 2016 (24)**	Meta-analysis of RCTs (68, 46 in cardiac surgery)	Cardiac and non- cardiac surgery (7,104 -4,840 cardiac surgery patients)	VA vs. TIVA (mainly propofol)	In cardiac surgery, VAs were associated with: - reduced overall mortality (either 30-day, 180-day or 1-year mortality) - reduced rate of pulmonary complications (effect more pronounced with sevoflurane and desflurane) No differences in hospital and ICU LOS	No benefit in non-cardiac surgery
**Likhvantsev, 2016 (19)**	RCT	On-pump elective CABG surgery (868)	Sevoflurane vs. TIVA	Sevoflurane was associated with: - reduced postoperative levels of cTn and NT-proBNP - reduced hospital LOS - reduced 1-year mortality (17.8% vs.24.8%)	Sevoflurane was administered continuously but without a pre- or postconditioning strict protocol
**El Dib, 2017 (25)**	Meta-analysis of RCTs (58)	On-pump and of- pump CABG (6,105)	VA vs. TIVA	Sevoflurane compared with propofol was associated with: - reduced 180-day to 1-year mortality - reduced postoperative use of inotropes and vasopressors	Some evidence of benefit on cardiac index for sevoflurane and desflurane
**Jensen, 2017 (26)**	Cohort study and propensity score matching	Cardiac surgery (17,771)	Sevoflurane vs. TIVA	No differences in 30-day mortality PMI, CKMB level and new dialysis No differences in long time stay in ICU, new ischaemic event and mortality within 6-months after the procedure	Study presented as an abstract only
**Landoni, 2019 (28)**	Multicentre RCT	Elective isolated CABG surgery (5,400, 64% on pump)	Anaesthetic regi- men including VA vs. TIVA	No significant difference in 1-year mortality (2.8% vs.3%). No significant differences in any of the secondary endpoints, such as 30-day mortality (1.4% vs.1.3%), and a composite of nonfatal PMI at 30 days or death at 30 days, death from cardiac causes at 30 days and at 1 year, hospital readmission during follow-up, and ICU and in-hospital LOS	Pragmatic, single-blind controlled trial at 36 centres in 13 countries There were three different strategies for VA administration At interim analysis, the trial was stopped for futility Did not mandate postoperative measurement of cTn
**Jiao, 2020 (29)**	Meta-analysis of RCTs and TSA (89)	CABG (mostly on pump) (14,387)	VA vs. TIVA	No significant differences in operative mortality, 1-year mortality or any of the postopera- tive safety outcomes. VAs reduce ICU and hospital LOS	TSA revealed that the results for operative mortality, 1-year mortality, LOS in the ICU, heart failure, stroke and the use of IABP were inconclusive
**Bonanni, 2020 (30)**	Meta-analysis of RCTs (42)	On-pump cardiac surgery (8,197)	VA vs. TIVA (mainly propofol)	VAs were associated with: - lower 1-year mortality (5.5% vs. 6.8%) - lower rate of PMI - lower cTn release - less need for inotropic medications - shorter time to extubation - higher cardiac index/output. No differences in short-term mortality (1.63% vs. 1.65%) and acute kidney injury	Studies on preconditioning or postconditioning only were excluded

Abbreviations: CABG, coronary artery bypass graft; CKMB, creatine kinase membrane isoform; ICU, intensive care unit; LOS, length of stay; MV, mechanical ventilation; NT-proBNP, N-terminal pro-brain natriuretic peptide; PMI, postoperative myocardial infarction; RCT, randomised controlled trial; TIVA, total intravenous anaesthesia; cTn, cardiac troponin; TSA, trial sequential analysis; VA, volatile anaesthetic; vs., versus.

Most probably, perioperative outcome of cardiac surgery patients depends more on how anaesthesiologists are capable of using the available tools and anaesthetic agents to control intra- and postoperative homeostasis in their patients.[31]

On the other hand, from the occupational health and climate change perspectives, TIVA seems to be preferable to inhaled anaesthetics (especially desflurane and nitrous oxide),[33] and we should also consider these factors when deciding the anaesthetic plan.[34]

## Conclusions

VA conditioning is fascinating, but its legacy is uncertain. [6] Although it is well established that the release of cardiac troponins is decreased by VA in cardiac surgery with CPB, favourable effects on mortality and major complications rates that were shown in small investigations and meta-analyses have not been confirmed in subsequent large multicentre RCTs. At present, there is no clear superiority of VA over intravenous anaesthesia in improving the outcome after cardiac surgery. Further large studies are needed to assess the role of anaesthetic regimen in cardiac surgery patients with high risk for myocardial ischaemia.
